# New existence results for nonlinear delayed differential systems at resonance

**DOI:** 10.1186/s13660-018-1912-7

**Published:** 2018-11-16

**Authors:** Ruipeng Chen, Xiaoya Li

**Affiliations:** Department of Mathematics, North Minzu University, Yinchuan, P.R. China

**Keywords:** 34B15, Positive periodic solution, Existence, Delay, Resonance, Fixed point

## Abstract

This paper deals with the first-order delayed differential systems

$$\textstyle\begin{cases} u'+a(t)u=h(t)v+f(t,u(t-\tau (t))), \\ v'+b(t)v=g(t,u(t-\tau (t))), \end{cases} $$

where *a*, *b*, *τ*, *h* are continuous *ω*-periodic functions with $\int_{0}^{\omega }a(t)\,dt=0$ and $\int_{0}^{\omega }b(t)\,dt>0$; $f\in C(\mathbb{R}\times [0,\infty ),\mathbb{R})$ and $g\in C( \mathbb{R}\times [0,\infty ),[0,\infty ))$ are *ω*-periodic with respect to *t*. By means of the fixed point theorem in cones, several new existence theorems on positive periodic solutions are established. Our main results enrich and complement those available in the literature.

## Introduction

In the past few decades, there has been considerable interest in the existence of positive periodic solutions of the first-order delayed equation

1.1 $$ u'+a(t)u=\lambda b(t)f\bigl(u\bigl(t-\tau (t)\bigr) \bigr), $$

where $a, b\in C(\mathbb{R},[0,\infty ))$ are *ω*-periodic with

$$\int_{0}^{\omega }a(t)\,dt>0,\quad\quad \int_{0}^{\omega }b(t)\,dt>0, $$

and *τ* is a continuous *ω*-periodic function. Note that when $\lambda =0$, equation (1.1) reduces to $u'=-a(t)u$, which is well known in Malthusian population models. In real world applications, (1.1) has also been viewed as a model for a variety of physiological processes and conditions including production of blood cells, respiration and cardiac arrhythmias. We refer the reader to [1–10] for some research work on this topic. Meanwhile, many authors have paid attention to the corresponding differential systems of (1.1), namely,

$$u'_{i}+a_{i}(t)u_{i}=\lambda b_{i}(t)f_{i}(u_{1},u_{2}, \ldots,u_{n}), \quad i=1,2,\ldots,n, $$

where $a_{i}, b_{i}\in C(\mathbb{R},[0,\infty ))$ are *ω*-periodic functions, and also supposed to satisfy

$$\int_{0}^{\omega }a_{i}(t)\,dt>0,\quad\quad \int_{0}^{\omega }b_{i}(t)\,dt>0, \quad i=1,2, \ldots,n. $$

See, for instance, Wang [11, 12], Chen et al. [13] and the references therein.

Obviously, the basic assumption $\int_{0}^{\omega }a(t)\,dt>0$ or $\int_{0}^{\omega }a_{i}(t)\,dt>0$ ($i=1,2,\ldots,n$), employed usually to guarantee the linear boundary value problem

1.2 $$ u'+a(t)u=0,\quad\quad u(0)=u(\omega ) $$

is non-resonant, has played a key role in the arguments of the above-mentioned papers. Indeed, it ensures a number of tools, such as fixed point theory, bifurcation theory and so on, could be applied to study the corresponding problems and establish desired existence results. Here (1.2) is called non-resonant if the unique solution of it is the trivial one. Moreover, if (1.2) is non-resonant then, provided that *h* is a $L^{1}$-function, the Fredholm alternative theorem implies the nonhomogeneous problem

$$u'+a(t)u=h(t),\quad\quad u(0)=u(\omega ) $$

always admits a unique solution, which can be written as

$$x(t)= \int_{0}^{\omega }G(t,s)h(s)\,ds, $$

where $G(t,s)$ is the Green’s function associated to (1.2), see [7–13] for more details.

Compared with the non-resonant problems, the research of the resonant problems proceeds very slowly and the related results are relatively little. Therefore inspired by the existing literature, we study the existence of positive periodic solutions of the following first-order delayed differential system:

1.3 $$ \textstyle\begin{cases} u'+a(t)u=h(t)v+f(t,u(t-\tau (t))), \\ v'+b(t)v=g(t,u(t-\tau (t))). \end{cases} $$

A natural and interesting question is whether or not (1.3) possesses a positive periodic solution provided that

1.4 $$ \int_{0}^{\omega }a(t)\,dt=0,\quad\quad \int_{0}^{\omega }b(t)\,dt>0, $$

which means *a* may change its sign on $\mathbb{R}$ and the linear differential operator $L_{a}u:=u'+a(t)u$ is resonant, $L_{b}u:=u'+b(t)u$ is non-resonant.

Recently, Domoshnitsky et al. [14] studied the following system of periodic functional differential equations:

1.5 $$\begin{aligned}& (M_{i}x) (t)\equiv x'_{i}(t)+\sum _{j=1}^{n}(B_{ij}x_{j}) (t)=f_{i}(t), \quad t\in [0,\omega ], i=1,2,\ldots,n, \end{aligned}$$

1.6 $$\begin{aligned}& x_{i}(0)=x_{i}(\omega )+c_{i}, \quad i=1,2, \ldots,n, \end{aligned}$$

where $x=\operatorname{col}(x_{1},\ldots,x_{n})$, $B_{ij}: C[0,\omega ] \to L[0,\omega ]$ are linear bounded operator for $i,j=1,\ldots,n$, $f_{i}\in L[0,\omega ]$ and $c_{i}\in \mathbb{R}$ for $i=1,\ldots,n$. Note that if the corresponding homogeneous problem

1.7 $$\begin{aligned}& (M_{i}x) (t)=0, \quad t\in [0,\omega ], i=1,2,\ldots,n, \end{aligned}$$

1.8 $$\begin{aligned}& x_{i}(0)=x_{i}(\omega ), \quad i=1,2,\ldots,n \end{aligned}$$

has only trivial solution, then, for any $f=\operatorname{col}(f_{1},\ldots,f _{n})$ and $c=\operatorname{col}(c_{1},\ldots,c_{n})$, system (1.5), (1.6) admits a unique solution *x* defined as [15]

1.9 $$ x(t)= \int_{0}^{\omega }G(t,s)f(s)\,ds+X(t)c, \quad t\in [0, \omega ]. $$

Here $G(t,s)$ is a $n\times n$ matrix and is called Green’s matrix of (1.5), the $n\times n$ matrix $X(t)$ is the fundamental matrix of (1.7) satisfying $X(0)-X(\omega )=E$, where *E* denotes the unit $n\times n$ matrix. By an inspection of (1.9), It is not difficult to see all properties of solutions are determined by $G(t,s)$ and $X(t)$. Hence a series of excellent results on positivity and negativity of $G(t,s)$ have been established in [14]. For instance, there are results on positivity (negativity) of $G(t,s)$ when all nondiagonal operators $B_{ij}$ ($i\neq j$, $i,j=1,\ldots,n$) are negative (positive), and results about positivity or negativity of the elements in the *n*th row of $G(t,s)$. To prove these results, the main idea and approach adopted by Domoshnitsky is to construct a corresponding first-order scalar functional equation

$$x'_{n}(t)+(Bx_{n}) (t)=f^{\ast }(t),\quad t\in [0,\omega ], $$

for *n*th component of a solution vector, here $B: C[0,\omega ]\to L[0, \omega ]$ is a linear continuous operator and $f^{\ast }\in L[0, \omega ]$. This idea has also been applied by Domoshnitsky to a study of the Cauchy problem [16] and some two-point boundary value problems [17].

In Sects. 2 and 3 of the present paper, the key idea of [14] to use non-resonant second scalar equation to obtain the assertion of $v(t)$, and then to set its representation in the first equation, will be employed to establish new existence theorems for system (1.3). To the best of our knowledge, the above-mentioned problem has not been studied so far and our results shall fill this gap. For the simplicity of statement, let *E* be the Banach space composed of continuous *ω*-periodic functions with the norm $\Vert u\Vert =\max_{t\in [0,\omega ]}\vert u(t)\vert $. For $q\in E$, we say $q\gg 0$ if it is strictly positive on $[0,\omega ]$, and $q\succ 0$ if *q* is nonnegative and $\int_{0}^{\omega }g(t)\,dt>0$. We denote by *q̄* and $\underline{q}$ the maximum and minimum of $q\gg 0$ on $[0,\omega ]$. By a positive *ω*-periodic solution of (1.3), we mean a vector function $(u,v)$, such that $u, v\gg 0$ are continuously differentiable everywhere and satisfy (1.3). Hence for the above question, if we choose $\chi \gg 0$ such that $p:=a+\chi \succ 0$, then $\int_{0}^{\omega }p(t)\,dt>0$, so the linear differential operator $Lu:=u'+p(t)u$ is invertible. In the following, we always assume $\tau \in C(\mathbb{R},\mathbb{R})$ is *ω*-periodic, and:

1. $a\in C(\mathbb{R},\mathbb{R})$ is *ω*-periodic with $\int_{0}^{\omega }a(t)\,dt=0$ and $b\succ 0$, $h\succ 0$;
2. there exists $\chi \gg 0$ such that $p=a+\chi \succ 0$;
3. $f\in C(\mathbb{R}\times [0,\infty ),\mathbb{R})$ is *ω*-periodic with respect to *t* and $f(t,u)\geq -\chi (t)u$;
4. $g\in C(\mathbb{R}\times [0,\infty ),[0,\infty ))$ is *ω*-periodic with respect to *t*.

### Remark 1.1

Note that *a* and *f* are assumed to be sign-changing, and therefore system (1.3) is more general than corresponding ones studied in the existing literature. For other research work on nonlinear differential equations at resonance, we refer the reader to [18–21] and the references listed therein.

The rest of the paper is arranged as follows. In Sect. 2, we introduce some preliminaries. And finally in Sect. 3, we state and prove our main results. In addition, several remarks will be given to demonstrate the feasibility of our main results.

## Preliminaries

Let $\tilde{G}(t,s)$ be the Green’s function of the linear boundary value problem

$$v'+b(t)v=0, \quad\quad v(0)=v(\omega ). $$

Then simple calculation gives the following.

### Lemma 2.1

*Let* (H1) *hold and* $\tilde{\delta }=e^{-\int _{0}^{\omega }b(t)\,dt}$. *Then*

$$\tilde{G}(t,s)=\frac{e^{\int_{t}^{s} b(\theta )\,d\theta }}{ \tilde{\delta }^{-1}-1},\quad t\leq s\leq t+\omega , $$

*and*

$$\tilde{m}:=\frac{1}{\tilde{\delta }^{-1}-1}\leq \tilde{G}(t,s)\leq \frac{ \tilde{\delta }^{-1}}{\tilde{\delta }^{-1}-1}=: \tilde{M}, \quad t \leq s\leq t+\omega . $$

By Lemma 2.1 and (H4), it is not difficult to verify

$$\textstyle\begin{cases} v'+b(t)v=g(t,u(t-\tau (t))), \quad t\in (0,\omega ), \\ u(0)=u(\omega ), \end{cases} $$

is equivalent to the equation

2.1 $$ v(t)= \int_{t}^{t+\omega }\tilde{G}(t,s)g\bigl(s,u\bigl(s-\tau (s) \bigr)\bigr)\,ds=:Au(t), $$

and $A:E\to E$ is completely continuous. Then system (1.3) can be equivalently written as the following integral-differential equation:

$$u'+a(t)u=h(t)Au(t)+f\bigl(t,u\bigl(t-\tau (t)\bigr)\bigr). $$

Moreover, by (H2) we get

2.2 $$ u'+p(t)u=\chi (t)u+h(t)Au(t)+f\bigl(t,u\bigl(t-\tau (t)\bigr) \bigr). $$

Clearly, if *u* is a positive *ω*-periodic solution of (2.2), then the original system (1.3) admits a positive *ω*-periodic solution $(u, v)$. In the following, we shall focus on studying (2.2).

Recall that (H2) ensures the linear differential operator $Lu:=u'+p(t)u$ is invertible. Therefore, by an argument similar to obtain Lemma 2.1, the Green’s function of

$$u'+p(t)u=0, \quad\quad u(0)=u(\omega ), $$

can be expressed as

$$K(t,s)=\frac{e^{\int_{t}^{s} p(\theta )\,d\theta }}{\delta^{-1}-1},\quad t\leq s\leq t+\omega , $$

where $\delta =e^{-\int_{0}^{\omega }p(t)\,dt}$, and accordingly,

$$m:=\frac{1}{\delta^{-1}-1}\leq K(t,s)\leq \frac{\delta^{-1}}{\delta ^{-1}-1}=:M, \quad t\leq s\leq t+ \omega . $$

Consequently, (2.2) can be written as the equivalent operator equation

$$u(t)= \int_{t}^{t+\omega }K(t,s) \bigl(\chi (s)u(s)+h(s)Au(s)+f \bigl(s,u\bigl(s- \tau (s)\bigr)\bigr) \bigr)\,ds=:Tu(t). $$

Setting $\sigma :=\frac{m}{M}$ and define

$$\mathcal{P}= \bigl\{ u\in E: u(t)\geq \sigma \Vert u \Vert , t\in [0,\omega ] \bigr\} . $$

Then $\sigma <1$ and $\mathcal{P}$ is a positive cone in *E*.

### Lemma 2.2

*Let* (H1)*–*(H4) *hold*. *Then* $T(\mathcal{P}) \subseteq \mathcal{P}$ *and* $T:\mathcal{P}\to \mathcal{P}$ *is compact and continuous*.

### Proof

Using (H3), and similarly to the proof of [12, Lemmas 2.2, 2.3] with obvious changes, we can easily get the conclusion. □

The following lemma is crucial to prove our main results.

### Lemma 2.3

(Guo–Krasnoselskii’s fixed point theorem [22, 23])

*Let* *E* *be a Banach space*, *and let* $\mathcal{P}\subseteq E$ *a cone*. *Assume* $\varOmega_{1}$, $\varOmega_{2}$ *are two open bounded subsets of* *E* *with* $0\in \varOmega_{1}$, $\bar{\varOmega }_{1}\subseteq \varOmega_{2}$, *and let* $T:\mathcal{P}\cap (\bar{\varOmega }_{2}\setminus \varOmega_{1})\to \mathcal{P}$ *be a completely continuous operator such that*

(i) $\Vert Tu\Vert \leq \Vert u\Vert $, $u\in \mathcal{P}\cap \partial \varOmega _{1}$, *and* $\Vert Tu\Vert \geq \Vert u\Vert $, $u\in \mathcal{P}\cap \partial \varOmega _{2}$; *or*
(ii) $\Vert Tu\Vert \geq \Vert u\Vert $, $u\in \mathcal{P}\cap \partial \varOmega _{1}$, *and* $\Vert Tu\Vert \leq \Vert u\Vert $, $u\in \mathcal{P}\cap \partial \varOmega _{2}$.

*Then* *T* *has a fixed point in* $\mathcal{P}\cap (\bar{\varOmega } _{2}\setminus \varOmega_{1})$.

## Main results

In this section, we shall state and prove our main findings. First we give the following notations:

$$\begin{aligned}& f_{0}=\lim_{u\to 0+}\frac{f(t,u)}{u}, \quad\quad f_{\infty }= \lim_{u\to \infty }\frac{f(t,u)}{u},\quad \text{uniformly for } t \in [0,\omega ]; \\& g_{0}=\lim_{u\to 0+}\frac{g(t,u)}{u}, \quad\quad g_{\infty }= \lim_{u\to \infty }\frac{g(t,u)}{u}, \quad \text{uniformly for } t \in [0,\omega ]. \end{aligned}$$

### Theorem 3.1

*Let* (H1)*–*(H4) *hold*. *If* $g_{0}=0$, $f_{\infty }=\infty $ *and*

3.1 $$ \lim_{u\to 0+}\frac{f(t,u)}{u}=-\chi (t), $$

*then* (1.3) *admits at least one positive* *ω*-*periodic solution*.

### Proof

For $0< r< R<\infty $, setting

$$\varOmega_{1}=\bigl\{ u\in E: \Vert u \Vert < r\bigr\} , \quad\quad \varOmega_{2}=\bigl\{ u\in E: \Vert u \Vert < R\bigr\} , $$

then we have $0\in \varOmega_{1}$, $\bar{\varOmega }_{1}\subseteq \varOmega_{2}$.

It follows from (3.1) that there exists $r_{1}>0$, such that, for any $0< u\leq r_{1}$,

$$f(t,u)\leq cu-\chi (t)u, $$

where *c* is a positive constant small such that $cM\omega \leq \frac{1}{2}$. Therefore, for $u\in \mathcal{P}$ with $\Vert u\Vert \leq r_{1}$, we can obtain

$$f(t,u)+\chi (t)u\leq cu, \quad t\in [0,\omega ]. $$

Moreover, since $g_{0}=0$, there exists $r_{2}>0$ so that $g(t,u) \leq du$ for any $0< u\leq r_{2}$. Thus, for $u\in \mathcal{P}$ with $\Vert u\Vert \leq r_{2}$, simple estimation gives

$$Au(t)= \int_{t}^{t+\omega }\tilde{G}(t,s)g\bigl(s,u\bigl(s-\tau (s) \bigr)\bigr)\,ds\leq \omega \tilde{M}\,d \Vert u \Vert , $$

where *d* is a positive constant small enough such that $\omega \,dM \tilde{M}h_{0}\leq \frac{1}{2}$ and $h_{0}=\int_{0}^{\omega }h(t)\,dt$. Let $r=\min \{r_{1}, r_{2}\}$. Then, for $u\in \mathcal{P}$ with $\Vert u\Vert =r$, we get

$$\begin{aligned} Tu(t) & = \int_{t}^{t+\omega }K(t,s) \bigl(\chi (s)u(s)+f\bigl(s,u \bigl(s-\tau (s)\bigr)\bigr) \bigr)\,ds+ \int_{t}^{t+\omega }K(t,s)h(s)Au(s)\,ds \\ &\leq cM\omega \Vert u \Vert + \omega \,dM\tilde{M}h_{0} \Vert u \Vert \leq \Vert u \Vert , \end{aligned} $$

which implies $\Vert Tu\Vert \leq \Vert u\Vert $, $\forall u\in \mathcal{P}\cap \partial \varOmega_{1}$.

On the other hand, $f_{\infty }=\infty $ shows there exists $\tilde{R}>0$ such that, for any $u\geq \tilde{R}$,

$$f(t,u)\geq \eta u, $$

where $\eta >0$ is a constant large enough such that $\sigma m\omega (\eta +\underline{\chi })\geq 1$ and $\underline{\chi }=\min_{t\in [0,\omega ]}\chi (t)$. Fixing $R>\max \{r, \frac{ \tilde{R}}{\sigma }\}$ and let $u\in \mathcal{P}$ with $\Vert u\Vert =R$, then

$$u(t)\geq \sigma \Vert u \Vert =\sigma R>\tilde{R} $$

and

$$f(t,u)+\chi (t)u\geq \eta u+\chi (t)u\geq \sigma (\eta +\underline{ \chi }) \Vert u \Vert ,\quad t\in [0,\omega ]. $$

Consequently, for $u\in \mathcal{P}$ with $\Vert u\Vert =R$, by (H4) we can obtain

$$\begin{aligned} Tu(t) & = \int_{t}^{t+\omega }K(t,s) \bigl(\chi (s)u(s)+f\bigl(s,u \bigl(s-\tau (s)\bigr)\bigr) \bigr)\,ds+ \int_{t}^{t+\omega }K(t,s)h(s)Au(s)\,ds \\ &\geq \sigma m\omega (\eta +\underline{\chi }) \Vert u \Vert \geq \Vert u \Vert . \end{aligned} $$

Hence $\Vert Tu\Vert \geq \Vert u\Vert $, $\forall u\in \mathcal{P}\cap \partial \varOmega _{2}$.

By Lemma 2.3(i), *T* has a fixed point $u^{\ast }\in \mathcal{P} \cap (\bar{\varOmega }_{2}\setminus \varOmega_{1})$, which is just a positive *ω*-periodic solution of (2.2). Subsequently, (1.3) admits at least one positive *ω*-periodic solution. □

### Theorem 3.2

*Let* (H1)*–*(H4) *hold*. *If* $f_{0}=\infty $, $g_{\infty }=0$ *and*

3.2 $$ \lim_{u\to +\infty }\frac{f(t,u)}{u}=-\chi (t), $$

*then* (1.3) *admits at least one positive* *ω*-*periodic solution*.

### Proof

We follow the same strategy and notations as in the proof of Theorem 3.1. Firstly, we show for $r>0$ sufficiently small,

3.3 $$ \Vert Tu \Vert \geq \Vert u \Vert , \quad \forall u\in \mathcal{P}\cap \partial \varOmega_{1}. $$

It follows from $f_{0}=\infty $ that there exists $\tilde{r}>0$ such that $f(t,u)\geq \beta u$ for any $0< u\leq \tilde{r}$, where $\beta >0$ is a constant large enough such that $\sigma m\omega ( \beta +\underline{\chi })\geq 1$. Therefore, for $0< r\leq \tilde{r}$, if $u\in \mathcal{P}$ and $\Vert u\Vert =r$, then

$$f(t,u)+\chi (t)u\geq \beta u+\chi (t)u\geq \sigma (\beta +\underline{ \chi }) \Vert u \Vert , \quad t\in [0,\omega ], $$

and

$$\begin{aligned} Tu(t) & = \int_{t}^{t+\omega }K(t,s) \bigl(\chi (s)u(s)+f\bigl(s,u \bigl(s-\tau (s)\bigr)\bigr) \bigr)\,ds+ \int_{t}^{t+\omega }K(t,s)h(s)Au(s)\,ds \\ &\geq \sigma m\omega (\beta +\underline{\chi }) \Vert u \Vert \geq \Vert u \Vert . \end{aligned} $$

Thus, (3.3) is true.

Secondly, we show for $R>0$ sufficiently large,

3.4 $$ \Vert Tu \Vert \leq \Vert u \Vert ,\quad \forall u\in \mathcal{P}\cap \partial \varOmega_{2}. $$

It follows from (3.2) that there exists $\tilde{R}>0$ so that

$$f(t,u)\leq \mu u-\chi (t)u $$

for any $u\geq \tilde{R}$, where $\mu >0$ satisfies $\mu M\omega \leq \frac{1}{2}$. Let $R_{1}>\max \{\tilde{r}, \frac{\tilde{R}}{ \sigma }\}$, then if $u\in \mathcal{P}$ and $\Vert u\Vert \geq R_{1}$, we get

$$u(t)\geq \sigma \Vert u \Vert \geq \sigma R_{1}>\tilde{R}, $$

and then

$$f(t,u)+\chi (t)u\leq \mu u\leq \mu \Vert u \Vert , \quad t\in [0,\omega ]. $$

Moreover, $g_{\infty }=0$ implies there exists $R_{2}>0$ so that $g(t,u)\leq \gamma u$ for any $u\geq R_{2}$ and $t\in [0,\omega ]$. Therefore, for $u\in \mathcal{P}$ with $\Vert u\Vert \geq R_{2}$, we have

$$Au(t)= \int_{t}^{t+\omega }\tilde{G}(t,s)g\bigl(s,u\bigl(s-\tau (s) \bigr)\bigr)\,ds\leq \omega \tilde{M}\gamma \Vert u \Vert , $$

where *γ* is a positive constant small enough such that $\omega \gamma M\tilde{M}h_{0}\leq \frac{1}{2}$. Let $R=\max \{R_{1}, R_{2}\}$. Then, for $u\in \mathcal{P}$ with $\Vert u\Vert =R$, we can obtain

$$\begin{aligned} Tu(t) & = \int_{t}^{t+\omega }K(t,s) \bigl(\chi (s)u(s)+f\bigl(s,u \bigl(s-\tau (s)\bigr)\bigr) \bigr)\,ds+ \int_{t}^{t+\omega }K(t,s)h(s)Au(s)\,ds \\ &\leq \mu M\omega \Vert u \Vert +\omega \gamma M\tilde{M}h_{0} \Vert u \Vert \leq \Vert u \Vert , \end{aligned} $$

which implies (3.4) is true.

Consequently, it follows from Lemma 2.3(ii) that *T* has a fixed point $u^{\ast }$ in $\mathcal{P}\cap (\bar{\varOmega }_{2}\setminus \varOmega _{1})$, which is just a positive *ω*-periodic solution of (2.2), Subsequently, (1.3) admits at least one positive *ω*-periodic solution. □

In the following, we investigate the multiplicity of positive *ω*-periodic solutions of system (1.3). To the end, we suppose:

(H5) $g_{0}=0=g_{\infty }$, and (3.1) and (3.2) hold. In addition, there exists $\alpha >0$, such that
  $$\min \bigl\{ f(t,u): \sigma \alpha \leq u\leq \alpha , t\in [0,\omega ]\bigr\} > \bigl( \mu -\sigma \chi (t)\bigr)\alpha , $$
  where $\mu >0$ satisfies $m\omega \mu \geq 1$.

### Theorem 3.3

*Let* (H1)*–*(H5) *hold*. *Then* (1.3) *admits at least two positive* *ω*-*periodic solutions*.

### Proof

Define

$$\varOmega_{3}=\bigl\{ u\in E: \Vert u \Vert < \alpha \bigr\} . $$

Let $\varOmega_{1}$ and $\varOmega_{2}$ be the same as in the proofs of Theorems 3.1 and 3.2. Then, for $0< r<\alpha <R$, we have $\bar{\varOmega }_{1}\subseteq \varOmega_{3}$, $\bar{\varOmega }_{3}\subseteq \varOmega_{2}$.

Applying $g_{0}=0$ and (3.1), and by an argument similar to the first part of the proof of Theorem 3.1, we can obtain

$$\Vert Tu \Vert \leq \Vert u \Vert , \quad \forall u\in \mathcal{P}\cap \partial \varOmega_{1}. $$

Similarly, combining $g_{\infty }=0$ and (3.2), we conclude

$$\Vert Tu \Vert \leq \Vert u \Vert , \quad \forall u\in \mathcal{P}\cap \partial \varOmega_{2}. $$

Obviously, the proof is finished if we prove

3.5 $$ \Vert Tu \Vert \geq \Vert u \Vert ,\quad \forall u\in \mathcal{P}\cap \partial \varOmega_{3}. $$

Suppose $u\in \mathcal{P}$ and $\Vert u\Vert =\alpha $, then $\sigma \alpha \leq \sigma \Vert u\Vert \leq u(t)\leq \Vert u\Vert =\alpha $, which together with (H5) yields

$$f(t,u)>\bigl(\mu -\sigma \chi (t)\bigr)\alpha , \quad t\in [0,\omega ], $$

and then

$$f(t,u)+\chi (t)u\geq f(t,u)+\sigma \chi (t)\alpha >\mu \alpha , \quad t \in [0, \omega ]. $$

Thus, similar to the first part of the proof of Theorem 3.2, we get

$$Tu(t)\geq m\omega \mu \alpha =m\omega \mu \Vert u \Vert \geq \Vert u \Vert , $$

and (3.5) is true. By Lemma 2.3, *T* has two fixed points $u_{1}$ and $u_{2}$, located in $\mathcal{P}\cap (\bar{\varOmega }_{3}\setminus \varOmega_{1})$ and $\mathcal{P}\cap (\bar{\varOmega }_{2}\setminus \varOmega _{3})$, respectively. Hence, (1.3) admits at least two positive *ω*-periodic solutions. □

Conversely, if (H5) is replaced with:

$(\mathrm{H}5)'$
$f_{0}=\infty =f_{\infty }$, and there exists $\alpha >0$ such that
  $$\begin{aligned}& \max \bigl\{ f(t,u): \sigma \alpha \leq u\leq \alpha , t\in [0,\omega ]\bigr\} < \bigl( \epsilon -\chi (t)\bigr)\alpha , \\& \max \bigl\{ g(t,u): \sigma \alpha \leq u\leq \alpha , t\in [0,\omega ]\bigr\} < \varepsilon \alpha , \end{aligned}$$
  where $\epsilon >0$, $\varepsilon >0$ satisfies $\epsilon M\omega \leq \frac{1}{2}$ and $\omega \varepsilon \tilde{M}Mh_{0}\leq \frac{1}{2}$, respectively, then similar to the proof of Theorems 3.1–3.3, we can prove the following.

### Theorem 3.4

*Let* (H1)*–*(H4) *and* $(\mathrm{H}5)'$ *hold*. *Then* (1.3) *admits at least two positive* *ω*-*periodic solutions*.

### Remark 3.1

We would like to point out that the results of Theorems 3.1–3.4 remain true for the special case $a(\cdot )\equiv 0$, i.e., for system

$$\textstyle\begin{cases} u'=h(t)v+f(t,u(t-\tau (t))), \\ v'+b(t)v=g(t,u(t-\tau (t))). \end{cases} $$

### Remark 3.2

It is worth remarking Theorems 3.1–3.4 apply to some equations which cannot be treated by the results of [7–10], and thus our main results are novel.

## Conclusions

We establish several novel existence theorems on positive periodic solutions for delayed differential systems (1.3), via fixed point theorem in cones. Our main findings Theorems 3.1–3.4 not only enrich and complement those available in the literature, but they also apply to some systems (equations) which cannot be dealt with by the results appeared in the existing literature.
